# The Comparative Effects of Myo-Inositol and Metformin Therapy on the Clinical and Biochemical Parameters of Women of Normal Weight Suffering from Polycystic Ovary Syndrome

**DOI:** 10.3390/biomedicines12020349

**Published:** 2024-02-02

**Authors:** Aleksandra Gudović, Zoran Bukumirić, Milos Milincic, Miljan Pupovac, Mladen Andjić, Katarina Ivanovic, Svetlana Spremović-Rađenović

**Affiliations:** 1 Clinic for Gynecology and Obstetrics, University Clinical Center of Serbia, 11000 Belgrade, Serbia; sasagudovic@gmail.com (A.G.); milosmilincic@gmail.com (M.M.); miljanpupovac@gmail.com (M.P.); andjicmladen94@gmail.com (M.A.); ikatarina.1996@gmail.com (K.I.); 2 Faculty of Medicine, Institute for Medical Statistics and Informatics, University of Belgrade, 11000 Belgrade, Serbia; zoran.bukumiric@med.bg.ac.rs; 3 Faculty of Medicine, University of Belgrade, 11000 Belgrade, Serbia

**Keywords:** PCOS, metformin, myo-inositol, insulin resistance, PCOS phenotypes, hyperandrogenism

## Abstract

Background: Polycystic ovary syndrome (PCOS) is a multisystem reproductive–metabolic disorder and the most common endocrine cause of infertility. The objective of our study was to determine the influence of myo-inositol (MI) on insulin resistance (IR), menstrual cycle regularity, and hyperandrogenism in women suffering from PCOS with normal BMI and diagnosed IR. Methods: We performed a prospective randomized controlled trial (RCT) that included 60 participants with PCOS who had IR and a normal BMI. Two groups were formed. A group of thirty patients received MI, and thirty patients in the control group received metformin (MET). Results: A statistically significant reduction in the area under the curve (AUC) of insulin values during the oral glucose tolerance test (OGTT) was recorded in both examined groups after the applied therapy with MI and MET. The regularity of the menstrual cycle in both groups was improved in >90% of patients. A statistically significant decrease in androgenic hormones (testosterone, SHBG, free androgen index—FAI, androstenedione) was recorded in both groups and did not differ between the groups. Conclusions: Both MI and MET can be considered very effective in the regulation of IR, menstrual cycle irregularities, and hyperandrogenism in women with PCOS.

## 1. Introduction

Polycystic ovary syndrome (PCOS) is a multisystem reproductive–metabolic disorder in women, with global prevalence between 8 and 13% [1]. PCOS can be related to metabolic abnormalities, such as impaired glucose tolerance, insulin resistance (IR), and the disturbance of the lipid profile. It represents the most common endocrine disorder in the reproductive years of a woman and is the leading cause of infertility [2]. 

The diagnosis of PCOS and phenotypes is made using Rotterdam diagnostic criteria [3]. Taking into account the heterogeneity of this syndrome, the Rotterdam criteria defines different phenotypes of PCOS: phenotype A or classic PCOS, characterized by hyperandrogenism, ovulatory dysfunction, and polycystic ovarian morphology; phenotype B characterized by hyperandrogenism and ovulatory dysfunction; phenotype C, comprising hyperandrogenism and polycystic ovarian morphology; and phenotype D, comprising ovulatory dysfunction and polycystic ovarian morphology [3]. The most common are phenotypes A 54%, B 8%, C 34%, and D 3% [4].

The role of insulin resistance in the development of PCOS has been extensively studied, and it is widely accepted that IR is an independent factor apart from obesity that plays a significant role in the molecular mechanisms leading to increased androgen synthesis in the ovary [5]. This is based on evidence showing a reduction in fasting insulin levels in women with PCOS who used insulin-sensitizing medications [6]. These women also experienced reduced androgen levels and improved ovarian function. Insulin receptors have been identified in the theca and granulosa cells of the ovary, clearly indicating that the ovary is a target organ for insulin action [7]. Hyperinsulinemia increases androgen production in the ovarian theca cells and reduces the production of SHBG in the liver, further favoring hyperandrogenemia and causing clinical features of hyperandrogenism—an irregular menstrual cycle, acne, excessive hair growth on areas of the body, thinning hair, and areas of darkened skin. 

Insulin resistance is present in 95% of obese women and in 75% of lean women with PCOS [4,8], although the degree of insulin resistance in women of normal weight with PCOS is usually significantly lower than in obese women with PCOS [9,10].

Therapeutic measures for PCOS include changes in lifestyle, medication, and surgical treatment [2]. This is particularly important for PCOS patients with a normal BMI and limited ability to correct insulin resistance through lifestyle changes. Among the various treatment modalities, two agents, metformin (MET) and myo-inositol (MI), have garnered substantial attention for their roles in managing PCOS. Both compounds exhibit distinct mechanisms of action and therapeutic effects, contributing to the amelioration of multiple PCOS-related facets.

One class of medication used in the treatment of insulin resistance in PCOS includes biguanides, mainly metformin. MET has been used as a staple in the treatment of type 2 diabetes mellitus (T2DM) for an extended period because of its ability to enhance the sensitivity of peripheral tissues to insulin and reduce its circulating levels [11]. Initially, MET was a pioneering insulin-sensitizing drug (ISD) utilized to explore the impact of insulin resistance on PCOS [12]. Multiple effects have been associated with MET in PCOS patients, including the restoration of ovulation, weight reduction, decreased androgen levels, a lowered risk of miscarriage, and reduced risk of gestational diabetes mellitus (GDM) [13]. Gastrointestinal symptoms, such as nausea, diarrhea, flatulence, bloating, anorexia, metallic taste, and abdominal pain, are the most commonly reported side effects. 

In recent times, MI has emerged as a safe alternative approach to therapy for patients with PCOS, both in cases of infertility and in younger individuals [14,15]. Recent studies suggest that the disruption in the insulin pathway might be due to a secondary messenger defect, inositol phosphoglycan, which plays a role in the activation of enzymes controlling glucose metabolism [16]. MI promotes GLUT4 translocation, inhibits adenylatecyclase, and reduces free fatty acid release. DCI, generated from MI under insulin stimulation, plays a role in glycogen storage and insulin signal transduction. MI and DCI may decrease insulin requirements by exerting an insulin-sensitizing effect, reflected in lower circulating insulin concentrations [17]. MI acts as an FSH secondary messenger, participating in FSH-mediated pathways that regulate granulosa cell proliferation and maturation. It modulates FSH-mediated AMH production, which is crucial for oocyte maturation, oviduct transport, and embryo quality. Tissues, including the ovaries, maintain a specific MI to DCI ratios, ensuring proper functionality. An imbalance in these ratios, with elevated DCI levels, may negatively impact oocyte and blastocyst quality. Both MI and DCI influence androgenic and estrogenic pools, potentially in opposite directions. DCI stimulates ovarian androgen production by theca cells and may downregulate estrogen synthesis [18]. The physiological ratios of MI to DCI vary in different tissues under normal and insulin resistance conditions. Altered inositol ratios may explain the imbalances in sex hormones observed in conditions like polycystic ovarian syndrome (PCOS) or due to pharmacological treatments, malabsorption, or dietary competition with glucose.

The objective of our study was to determine the influence of MI on insulin resistance, menstrual cycle regularity, and hyperandrogenism in women suffering from PCOS with normal BMI, and diagnosed IR, and to compare this to the effects of MET.

## 2. Materials and Methods

### 2.1. Patients

The study was conducted at the Clinic for Gynecology and Obstetrics of the University Clinical Center of Serbia, at the Department of Gynecological Endocrinology, in the period from June 2017 to June 2018. This study was prospective and randomized and included 80 normal-weight PCOS women, and insulin resistance treatment was randomized to 40 patients who received metformin (Glucophage, Merck, Rahway, NJ, USA) and 40 who received myo-inositol (Inofolic Lo.Li. Pharma R, Galaxy, Rome, Italy). Metformin therapy started at a dose of 500 mg/day during meals. The initial dose was increased after 3 weeks by another 500 mg to a total dose of 1500 mg/day, which was reached after 6 weeks from the start of drug administration. The effects of therapy were evaluated after 6 months of continuous administration of a dose of 1500 mg/day. All patients in this group were treated in the same way and with the same dose. The patients who were treated with Inofolic, from the start, took a prepared dose containing 2000 mg of myo-inositol and 200 mcg of folic acid twice a day. The effect of Inofolic therapy was assessed after 8 months of using this preparation.

**Figure 1:**
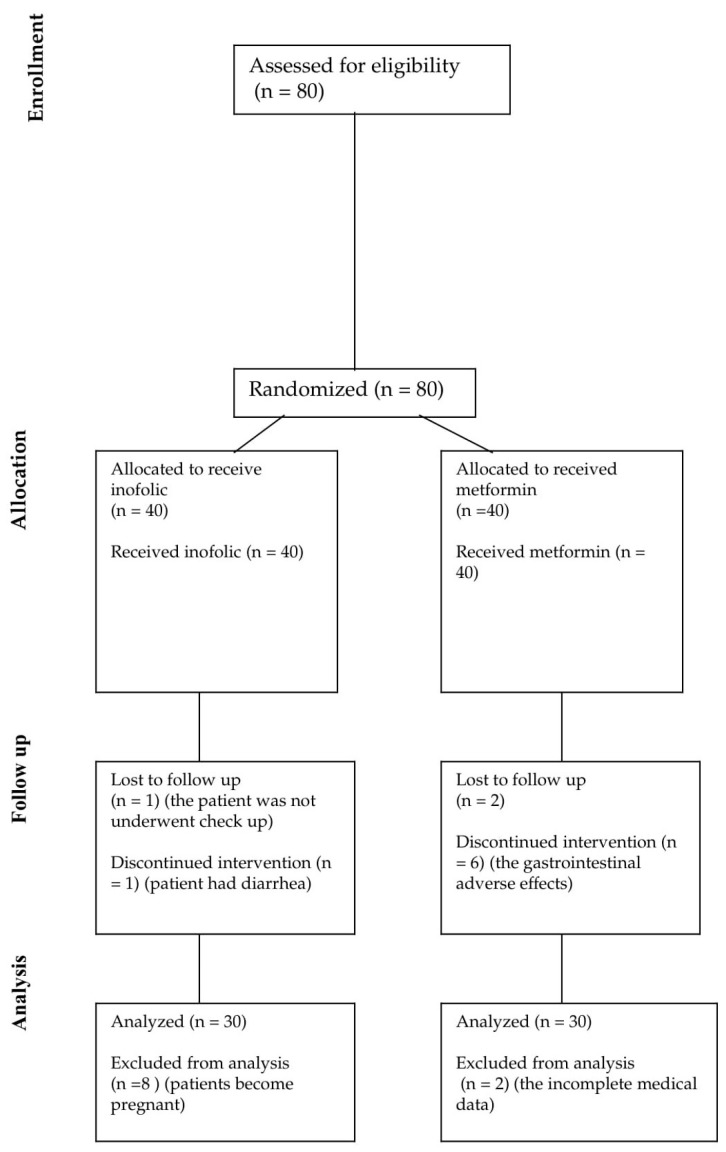
Follow-up chart of patients during the study.

The study’s inclusion criteria were as follows: over 18 years of age, confirmed diagnosis of PCOS according to the Rotterdam criteria, the presence of insulin resistance, and BMI in the range of 19–25 kg/m^2^ [3,19]. The exclusion criteria included the following: any acute and chronic diseases as well as the chronic use of the therapy, including medications, vitamins, and supplements. Data about patients were obtained from the anamnestic data, which were recorded in medical documentation. This study was conducted in accordance with the guidelines proposed in the Declaration of Helsinki. The research was approved by the Ethics Committee of the Faculty of Medicine, University of Belgrade (No:15-EK-05). All patients gave written consent to be included in the study. During the study, 10 patients from the Inofolic therapy group withdrew from the study because 8 of them became pregnant, 1 had nausea and gave up because of that, and 1 patient did not come for the check-up. Also, the 10 patients from the metformin therapy group withdrew from the study because 6 of them had gastrointestinal adverse effects and stopped with therapy, 2 did not come for the check-up, and for 2 patients, there were incomplete clinical data (Figure 1).

### 2.2. Study Protocol

The endocrinological examination was performed on the second day of the menstrual cycle, or if amenorrhea was present any day in the absence of dominant structures on the ovary, such as the corpus luteum or dominant follicle, visualized via an ultrasound examination. FSH, LH, estradiol, progesterone, prolactin, testosterone, androstenedione, DHEA-S, 17-OH progesterone, and SHBG from the blood were determined, and blood glucose and lipids were also measured. If blood glucose was less than 7 mmol/L, OGTT with insulinemia was performed. The day after taking basal analyses, the test subjects underwent a standard three-hour oral glucose tolerance test (OGTT) with 75 g of glucose after an overnight fast of at least 8 h. The ultrasound examination of the small pelvis was performed on a Siemens Sonoline Sienna device with a 3.75 MHz probe on the same day of blood sampling. The dimensions of the uterus, structure, and thickness of the endometrium, ovary structure, and volume were also measured. According to the results, patients with an established diagnosis of PCOS, normal BMI, and insulin resistance were randomly divided into two groups and treated with metformin or infolic.

#### 2.2.1. Diagnostic Criteria and Normal Values

PCOS was diagnosed according to the Rotterdam criteria, which implies that any 2 out of the following 3 exist: (1) hyperandrogenism (clinical and/or biochemical), (2) an ovulatory dysfunction (oligo- or anovulation), and (3) morphologically polycystic ovaries [4,20]. All of our patients were evaluated for the presence of other endocrinological disorders, such as hyperprolactinemia, thyroid function disorders, congenital adrenal hyperplasia, premature ovarian failure, Cushing’s syndrome, acromegaly, and androgen-secreting tumors. PCOS phenotypes were recorded as follows: phenotype A, hyperandrogenism (HA), chronic anovulation (ANOV) and morphological polycystic ovaries (PCOM); phenotype B, hyperandrogenism (HA) and chronic anovulation (ANOV); phenotype C, hyperandrogenism (HA) and morphological polycystic ovaries (PCOM); and phenotype D, chronic anovulation (ANOV) and morphologically polycystic ovaries (PCOM) [21].

Regular menstrual cycles were defined as those whose duration was 21 to 35 days. Amenorrhea was defined as the absence of menstrual bleeding for more than 6 months. Oligomenorrhea was defined as menstrual cycles ranging from 35 days to 6 months and an annual number of menstrual cycles less than 8. The regularity of the menstrual cycle of the patients in both groups was assessed as regular, irregular up to 3 months, irregular for 3–6 months, and irregular for more than 6 months. Biochemical hyperandrogenism was defined as the free androgen index (FAI) > 6 and/or a total testosterone serum concentration > 3.3 nmol/L and serum androstenedione concentration > 2.26 ng/mL. The free androgen index (FAI) was calculated using the formula FAI = (testosterone × SHBG)/100.

Insulin resistance was assessed using the homeostatic model of insulin resistance (HOMA-IR) and the following formula: fasting blood glucose × fasting insulin/22.5 [21]. The value of HOMA IR in healthy subjects is considered less than 2.5. Also, semi-quantitative criteria for basal, maximal and second-hour glycaemia during the OGTT were used to define insulin resistance based on the threshold values for insulin at 0 min (I0 > 22, 1 µIU/mL), 60 min (I60 > 130 µIU/mL) and at 120 min (I120 > 30 µIU/mL), which are given in Greenspan’s basic and clinical endocrinology [22]. Also, the area under the curve for glucose (AUC glucose) and insulin (AUC insulin) during the OGTT was determined according to the trapezoidal model as indices of insulin resistance/sensitivity derived from the OGTT and used for statistical analyses [23]. The morphology of polycystic ovaries was diagnosed using a transvaginal ultrasound examination in the presence of (1) 12 or more follicles (diameter 2 to 9 mm) in each ovary, (2) an ovarian volume greater than 10 mL, (3) a finding sufficient in just one ovary. The volume of the ovary was calculated from the following formula: thickness x width x height of the ovary in mm × 0.52. The transvaginal ultrasound examination was performed on the same day as the endocrinological examination.

#### 2.2.2. The Methods of Measuring Hormones Levels

The serum concentrations of FSH were determined immunoradiometrically (IRMA hFSH, INEP, Belgrade, Serbia, with intra- and inter-assay CV 5.56 and 9.52%). Reference values of FSH in the follicular phase were 2–15 IU/L. Serum concentrations of LH were determined immunoradiometrically (IRMA hLH), INEP, Belgrade, Serbia, with intra- and inter-assay CV 5.82 and 9.86%). Reference values of LH in the follicular phase were 1–10 IULL. Serum concentrations of prolactin were determined immunoradiometrically (IRMA hPRL), INEP, Belgrade, Serbia, with intra- and inter-assay CV 3.46 and 3.63%). Prolactin reference values were 59–619 mIU/L. Serum insulin concentrations were determined using a radioimmunoassay (RIA INSULIN (PEG), INEP, Belgrade, Serbia, with an intra- and inter-assay KV 2.5 and 7.7%]). Serum testosterone (nmol/L) was determined via radioimmunoassay (TESTO-CT2, Cisbio Bioassays, Codolet, France, with intra- and inter-assay CV of 3.1 and 5.2%). Testosterone reference values were 0.3–3.3 nmol/L.SHBG (nmol/L) and determined using a radioimmunoassay (SHBG-RIACT, Cisbio Bioassays, Codolet, France, with intra- and inter-assay CV of 5.2 and 5.3%). SHBG reference values were 18–87 nmol/L. DHEAS (μmol/L) was determined using a radioimmunoassay (DHEAS-RIA-CT, DIA source, Belgium, with intra- and inter-assay KV 3.6 and 6.5%). DHEAS reference values were 0.56 14.4 μmol/L. Androstenedione (ng/mL) was determined using a radioimmunoassay (R-GM-100, ZenTech, Angleur, Belgium, with an intra- and inter-assay CV of 8.7% and 3.7%). Androstenedione reference values were 0.02–2.26 ng/mL. 17-OH-progesterone was determined with (MP Biomedicals, Solon, OH, USA) an intra- and inter-assay (KV 8.3 and 12.8%). Reference values of 17-OH-progesterone in the follicular phase were 0.303–2.42 nmol/L. Estradiol was determined via a radioimmunoassay (ESTR-US-CT, Cisbio Bioassays, Codolet, France, with intra-species CVs of 5.0% and 9.7%). Reference values of estradiol in the follicular phase were 105–217 pmol/L. Serum concentrations of progesterone were determined using a radioimmunoassay (RIA PROGESTERON (PEG), INEP, Belgrade, Serbia, with intra- and inter-assay CV 5.62 and 5.67%). Reference values of progesterone in the follicular phase were 1.0–9.5 nmol/L. Glycemia (mmol/L) was determined using the glucose oxidase method (Randox, Crumlin Great Britain) and an auto-analyzer (Beckman, Vienna, Austria). Blood for glycemia and insulin analysis was taken at 0, 30, 60, 120, and 180 min of the test. 

The concentrations of FSH, LH, TSH, and prolactin in the serum were determined using the immunoradiometric method (IRMA). The principle of the test involves the use of two monoclonal antibodies for the different epitopes of the molecule of interest—the analysis. The analysis from the sample reacts simultaneously with the monoclonal antibody attached to the bottom of the test tube, and then the monoclonal antibody is labeled with radioactive 125 I. When the incubation is complete, the contents of the tube are aspirated to remove unbound components. The radioactivity of the complex that remains bound to the test tube is measured in a gamma counter. The amount of radioactivity measured is directly proportional to the concentration of the analysis in the sample.

The concentration of insulin, testosterone, androstenedione, SHBG, DHEAS, estradiol, and progesterone in the serum is determined using the RIA method. The test is based on the competitive binding of analyses from samples or standards and a radioactively labeled analysis derivative to a certain number of epitopes on specific antibodies, whereby labeled and unlabeled immune complexes are formed. The less labeled complex is formed if there are more analyses in the sample. After the reaction is complete, all resulting complexes are precipitated with an immunoadsorbent, which is a combination of secondary antibodies and polyethylene glycol (PEG), while the free analyze (labeled and unlabeled), as well as free antibodies, remain in the liquid phase. The radioactivity of the precipitate is measured with a suitable gamma scintillation counter. At the same time as serum samples, standards containing different precisely defined concentrations of molecules of interest are also treated, with the help of which a standard curve is formed. Analyzing the concentration in serum samples is determined by comparison with the standard curve.

The glucose concentration was determined using the glucose oxidase method. The principle of this method is that glucose is oxidized by the enzyme glucose oxidase to gluconic acid; meanwhile, the oxygen is reduced to hydrogen peroxide. The nascent oxygen, which forms from hydrogen oxidase, reacts with 4-aminoantipyrine, which further reacts with the phenol and produces quinoneimine, a colored compound whose color in the colorimetric analysis correlates with the concentration of the glucose in the sample.

### 2.3. Data Analysis Methods

Statistical analyses were performed using SPSS Ver. 13.0. A statistical test value of less than 0.05 (*p* < 0.05) was considered significant. Data were described via the following descriptive statistical methods: measures of central tendency, measures of variability (interval of variation, standard deviation, and interquartile range), and relative numbers. Data analysis involved the use of methods for assessing the significance of differences: Student’s *t*-test for unpaired samples, the Mann–Whitney U test, Chi-square test (χ^2^ test), Fisher’s exact probability test, Fisher’s analysis of variance (ANOVA) and Kruskal–Wallis analysis of variance, depending on the chosen measurement scale. Also, the following methods were used for assessing the significance of associations: Pearson’s linear correlation coefficient and Spearman’s rank correlation coefficient, as well as different types of single and multiple regression models.

## 3. Results

The follow-up chart of patients in our randomized study is shown in Figure 1.

In Table 1, the clinical and endocrinological characteristics of the patients in the studied groups before the initiation of therapy are shown.

There were no statistically significant differences in the mean values of age, BMI, or mean levels of the AMH, FSH, LH, estradiol, progesterone, prolactin, TSH, testosterone, androstenedione, DHEAS, FAI, SHBG, HOMA-IR and fasting insulin between the group which received gluformin and inositol before therapy, respectively. 

In our study groups, phenotype A was the most frequent in both groups of patients—66.7% (*n* = 20) among patients who received metformin and 63.3% (*n* = 19) among patients who received Inofolic. The second most frequent was phenotype D, in both groups of patients, at 26.7% (*n* = 8) and 23.3% (*n* = 7), respectively. The frequencies of phenotype B and phenotype C were 3.3% (*n* = 1 in each group) among patients in the gluformin group, respectively. On the other hand, the phenotype B and phenotype C frequencies were 6.7% (*n* = 2 in each group) in the Inofolic group, respectively. There was no statistically significant difference in the frequency of PCOS phenotypes between these two groups of patients (Fisher’s exact probability test; *p* = 1.000).

We observed a statistically significant decrease in the mean values of FSH, testosterone, androstenedione, DHEAS, FAI, SHBG, HOMA-IR, and fasting insulinaemia after therapy in both groups. Also, we observed no statistically significant decreases in the LH/FSH ratio in both groups. The values of the examined clinical parameters of the patients after therapy in both groups are shown in Table 2.

**Figure 2:**
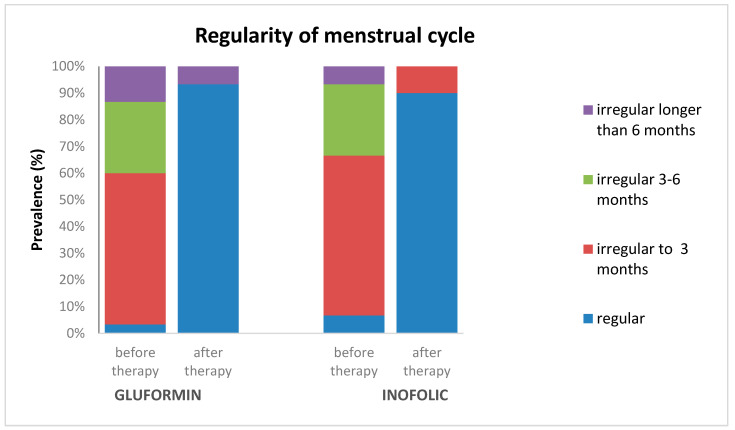
The regularity of the menstrual cycle in patients before and after treatment with gluformin or Inofolic R.

Also, the largest number of patients, 56.7% of them in the group that received glufomin and 60% of the patients who received Inofolic R, reported an irregularity in their menstrual cycle for a period of up to 3 months. Before starting the therapy, 3.3% of the patients who received Gluformin and 6.7% of patients who received Inofolic R had a regular menstrual cycle, which is not a statistically significant difference (Mann–Whitney U test, U = 404.0; *p* = 0.442). In total, 93.3% of patients who received Gluformin and 90.0% who received Inofolic R had a regular cycle after therapy, which was not a statistically significant difference (Mann–Whitney U test, U = 438.0; *p* = 0.711). In both groups of patients, there was a statistically significant difference in the regularity of cycles before and after the use of gluformin or Inofolic R (*t*-test, Z = 4.696, *p* < 0.001; *t*-test, Z = 4.724, *p* < 0.001), respectively. The regularity of the menstrual cycle in patients before and after treatment with gluformin or Inofolic is shown in Figure 2.

**Figure 3:**
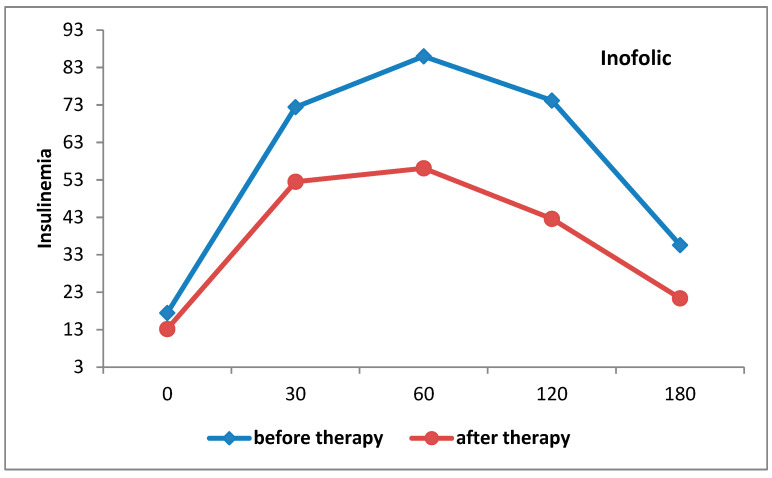
Insulinemia curves in patients treated with Inofolic R before and after therapy.

There was statistically significant decrease in the AUC of insulinaemia in the group that received InofolicR (F = 56.247; *p* < 0.001) (Figure 3). 

**Figure 4:**
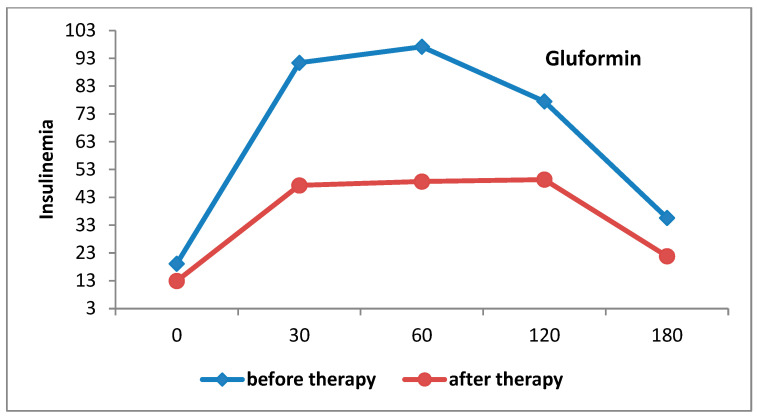
AUC of insulinaemia curves in patients treated with gluformin before and after therapy.

There was a statistically significant decrease in the AUC of insulinaemia in the group that received gluformin (F = 56.247; *p* < 0.001) (Figure 4). 

**Figure 5:**
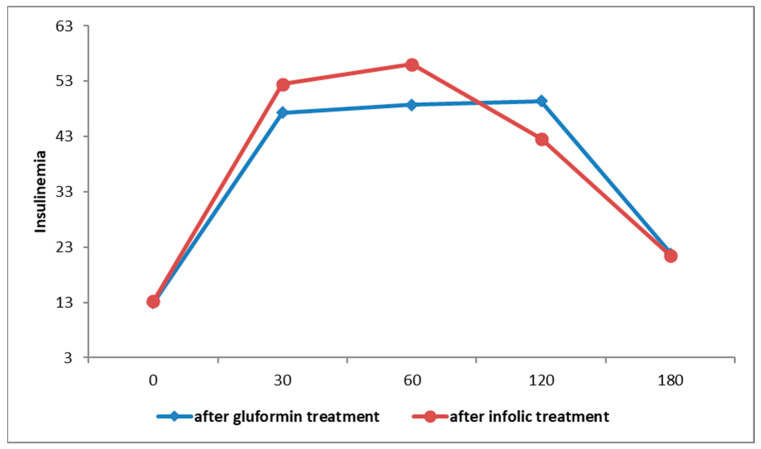
Insulinemia curves after therapy with gluformin or Inofolic R.

Although there was a statistically significant decrease in the insulin AUC value in both studied groups before and after the treatment with gluformin or Inofolic R, there was no statistically significant difference between the studied groups in AUC values of insulin after the applied therapies (F = 0.039; *p* = 0.843) (Figure 5).

**Figure 6:**
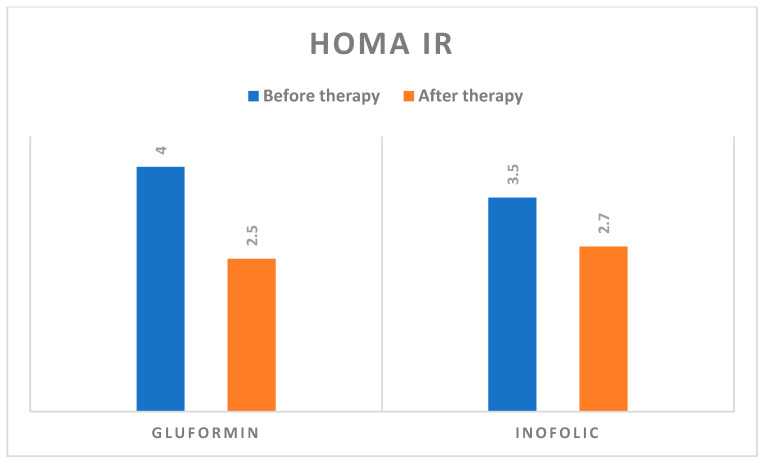
HOMA IR before and after treatment with gluformin or Inoflic R.

We also observed a statistically significant decrease in HOMA IR after either i = Inofolic R or gluformintherapy (F = 17,013; *p* < 0.001) (Figure 6).

Comparing phenotype A patients with phenotype D patients within the same therapy group (metformin or inofolic), a statistically significant lower AUC value of insulin was observed after metformin therapy in patients with phenotype A (*p* = 0.017). Also, phenotype A patients who were treated with inofolic had statistically significantly higher FAI values compared to phenotype D patients before starting therapy (*p* = 0.046). LH values were statistically and significantly higher in patients with phenotype A compared to patients with phenotype D in the metformin-treated group before starting therapy (*p* = 0.039).

If we compare patients with the same phenotype, depending on whether they used metformin or inofolic, it was observed that patients with phenotype A who used metformin had a statistically significant lower AUC of insulin after therapy compared to patients with phenotype A who used inofolic (*p* = 0.018). Phenotype A patients treated with inofolic had statistically significant higher FSH values before starting therapy compared to phenotype A patients receiving metformin (*p* = 0.037). Also, phenotype D patients from the inofolic group had statistically significantly higher LH values before starting therapy compared to phenotype D patients from the metformin group (*p* = 0.021).

## 4. Discussion

This randomized controlled trial (RCT) demonstrates a detailed comparison between two commonly used insulin sensitizers in the treatment of PCOS. The aim of this study was to compare the effects of myo-inositol (MI) and metformin (MET) on characteristic metabolic markers, the biochemical and clinical effects of androgens, and the menstrual cycle length in PCOS patients. If your sole objective is to assess the effectiveness of MI in isolation, it is advisable to incorporate a control group receiving only folic acid. However, due to ethical considerations, the inclusion of a placebo group with only folic acid was not deemed feasible. Moreover, our research aimed to delve into a comparative analysis of two established drugs for testing insulin resistance. The participants of both groups were well-matched in terms of age, BMI, and metabolic profile. Given the known impact of obesity on hyperinsulinemia and other metabolic and hormonal aspects, only subjects with a normal BMI (21.9 ± 2.9 vs. 21.6 ± 2.5, t = 0.514; *p* = 0.609) were included in the study. No statistically significant difference was found in the average BMI values between the examined groups before and after therapy with MI and MET (t = 0.519; *p* = 0.523). Different results were obtained by Ravn et al., where there was a decrease in BMI in the MET group but not in the MI group, although they performed a study on obese subjects with an average BMI of 34.5 [24]. In our study, six patients who had intense gastrointestinal disorders discontinued therapy and left the study, so it cannot be ruled out that they lost weight. Choosing women with a low BMI avoids the influence of obesity and reduces BMI on insulin resistance and endocrinological parameters [25]. In individuals who are obese, higher amounts of non-esterified fatty acids, glycerol, hormones, and pro-inflammatory cytokines that could participate in the development of insulin resistance are released by the adipose tissue [25]. In peripheral fatty tissue conversion to estrone, although it has weak levels of estrogen due to its amount, compensates for low potency and leads to the disruption of endocrine status. In our examined group of PCOS women with normal BMI, an improvement in the parameters was due to MET/MI therapy, not the impact of weight loss, which in our patients was excluded.

### 4.1. Metabolic Changes

Insulin resistance was proven in all subjects. For the clinical diagnosis of insulin resistance, we used the HOMA IR index with the addition of glucose and the insulin area under the curve (AUC) for more precise results and statistical analyses. The reasoning behind this was that Hba1c and fasting plasma glucose (FPG) are most often used to determine diabetes and glucose intolerance. However, they are not sufficient for the clear detection of glucose intolerance in the early stage. On the other hand, hyperglycemic/euglycemic clamp methods are time-consuming, labor-intensive, and expensive. Experienced personnel must be able to perform and supervise the tests, and human error can lead to the misrepresentation of graft function. 

Our results show statistically significant changes in HOMA-IR, as well as in AUC values after treatment in both MI and MET groups. There was no statistically significant difference in any parameter between the two treatment groups at the end of therapy. These findings provide valuable insights into the efficacy of both treatments in managing glucose and insulin levels.

Comparable results on the improvement of glucose–insulin parameters were found in the studies that compared MET vs. MI with a similar methodology of duration for the therapy, dosage of medications, and a number of subjects, while the BMI of participants was higher [26,27]. In the study by Shokrpour et al., the 12-week RCT comparing MI and MET in women with PCOS revealed that MI supplementation resulted in significant improvements in glycemic control, triglycerides, and VLDL–cholesterol levels compared to MET [28]. In the large meta-analysis of RCTs in the comparison of two treatments (MET and MI), there was no statistical difference between the two medications on the fasting insulin (*p* = 0.697), HOMA index (*p* = 0.635), testosterone (*p* = 0.922), SHBG levels (*p* = 0.263), and BMI (*p* = 0.265) [29]. This meta-analysis also clearly showed fewer side effects of MI compared to MET, which may be beneficial in patients who cannot tolerate MET.

In a comparable study (except for the BMI value (34.4 kg/m^2^)), MI and MET in women with PCOS found no significant effect on the primary outcome, HOMA-IR, with either treatment. However, positive effects on fasting blood glucose, weight reduction, and high-density lipoprotein (HDL) levels were observed with MET. Cycle length changes were comparable between MI and MET. Notably, adverse effects were less frequent with MI. Adverse effects appeared in four women during MI and 16 women during MET (MI vs. MET, *p* = 0.001) [24]. Unchanged HOMA-IR in MET vs. MI RCT studies was also in the findings of the following studies with comparable parameters to our study [30,31,32].

### 4.2. Changes in the Androgens

Hyperandrogenism is a key feature of PCOS, contributing to various clinical manifestations and potential long-term health implications, and is often associated with insulin resistance, which can lead to an increased risk of metabolic complications, including type 2 diabetes and cardiovascular disease. For these reasons we based a large portion of our research on the effects of therapy on the endocrine system, mainly the follicle-stimulating hormone (FSH), luteinizing hormone (LH), total testosterone (TT), SHBG, dehydroepiandrosterone sulfate (DHEAS), 17OH-progesterone (17-OHP), androstenedione, estradiol, insulin, fasting blood glucose (FBG), prolactin, TSH, free thyroxine (fT4), free androgen index (FAI), and the anti-Müllerian hormone (AMH). A statistically significant improvement in all hyperandrogenism parameters was observed, while no significant improvement in the LH/FSH ratio was observed. There was an increase in SHBG values in both groups. Our data are consistent with previous studies [33,34,35,36].

### 4.3. Menstrual Cycle Regularity

Effectiveness in regulating the cycle was shown by both medications in a significant percentage. At the beginning of the study, 6.7% of MI subjects had regular cycles compared to 3.3% in the MET group. At the end of the test, 90% and 93.3% had a regular cycle, respectively (*p* < 0.001). Our results suggest that there was no statistically significant difference in the frequency of regular menstrual cycles between subjects who used MET and MI in therapy. Both drugs can be considered effective in regulating the menstrual cycle in women with PCOS [12,29,30]. Research provides substantial evidence of the efficacy of both MET and MI in normalizing menstrual cycles in women with PCOS. These findings are consistent with existing studies, supporting the use of these treatments to address menstrual irregularities in PCOS patients.

### 4.4. Adverse Effects, Non-Completion, and Pregnancy

In the MI group, we achieved eight clinical pregnancies, which can be explained through the enhancing effect on the action of FSH. MI was found in follicular fluid, where its role in oocyte maturation has been confirmed [35]. It may provide valuable insights into the potential effects of MI on fertility. Also, the 10 patients from the MET therapy group withdrew from the study because 6 of them had gastrointestinal adverse effects, 2 did not come for the check-up, and 2 patients had incomplete clinical data. This was expected as MET is well known and seen to cause frequent GI side effects [36].

An analysis of the representation of phenotypes in our research showed that phenotypes A and D occur with the highest frequency. Subjects from the group who had MET in therapy most often had phenotype A, at 66.7%, as well as subjects who had MI at 63.3% in therapy. Phenotypes B and C were equally represented in both groups at 3.3%, and phenotype D in the first group was 26.7%, and the second group was 23.3%. It was possible to compare the results of phenotypic groups A and D. and we conclude that there is a difference between these groups in response to gluformin therapy, considering that group A shows a more significant decrease in the AUC during therapy than group D. The insulin response to inofolic during OGTT was similar in both phenotypic groups. When comparing the response of the phenotypic group to the two investigated drugs, phenotypic group A showed a more significant decrease in the AUC’s response to gluformin than to inofolic. Other parameters that could be examined—HOMA and FAI—did not show a difference in relation to the phenotypic group and the type of medication used. The largest number of women with PCOS had hyperandrogenism, which is 45 out of 60 women, considering the presence of phenotypes A, B, and C. These findings did not differ from other research papers on this subject [37].

Notably, some research has proposed that a combination of MET and MI may yield synergistic effects in PCOS management, addressing both the metabolic and hormonal aspects of the condition [38]. In the international evidence-based guideline for the evaluation and management of PCOS 2023, their pooled evidence suggests that MI has fewer GI effects than MET. MET was superior to MI for IR, and MI was superior to MET in cycle regulation [2].

The limitations of our work are that, due to the relatively small sample size, we did not compare results between each PCOS phenotype separately, which further shows how MET and MI work differently, especially compared to PCOS with elevated androgens and non-hyperandrogenic PCOS. It is difficult to compare the results of our study with other studies that are heterogeneous in terms of the composition of the drug containing inositol (only inositol and folic acid, MI plus DCI in different composition, MI and chiro inositol plus different vitamin D, vitamin C or saffron) and there are a lot of confounding components in this area of research. This group was also heterogeneous in regard to the participants, as some studies included both lean and obese patients with PCOS. Taking into account that our study population consisted of patients with a normal body mass index, all patients were advised to follow a healthy diet without special reductions. They were advised to avoid juices, carbonated drinks, concentrated sugars, white sugar, and white flour. Regarding the physical activity of the participants throughout the study, individuals who had previously engaged in physical activity maintained their established regimen, while those who had not been following any exercise routine did not initiate a new one during the course of the research.

Our study’s main novelty in PCOS treatment is that all subjects treated were of lean BMI (BMI < 22 kg/m^2^), and the medicament only contained inositol and folic acid, which is contrary to existing studies and had no confounding effect. We treated two study groups with the most commonly used insulin sensitizers for PCOS treatment—MET and MI. This study is one of the first prospective randomized studies related to studying the effects of MI. The unexpected outcome was that eight women became pregnant, which was not the primary goal of the study but indicated the significant impact of MI on fertility, which was confirmed in later studies in larger groups of PCOS patients.

## 5. Conclusions

The findings of our study provide valuable insights into the efficacy of both treatments—MET and MI—in managing the key pathophysiologic parameters of PCOS. We concluded that both MET and MI showed similar therapeutic efficiency and promising results in improving insulin resistance, reducing hyperandrogenemia, and improving menstrual regularity in women of normal weight suffering from PCOS. In the course of this study, the lower adherence of patients to metformin therapy due to gastrointestinal effects and a significant effect of inoflic on fertility was established, although this was not the primary goal of this research. Therefore, inofolic can be considered the first line of therapy in lean patients suffering from PCOS who are insulin resistant. The possibility of different responses to treatment with MET or inofolic in relation to the phenotypic group warrants further investigation.

## Figures and Tables

**Table 1:** The clinical and endocrinological characteristics of patients treated with gluforminorInofolic R before the initiation of therapy.

Variable (Unit)	Metformin (*n* = 30)	Inofolic (*n* = 30)	Statistical Significance (*p* Value)
Age (years)	28.0 ± 4.9	26.3 ± 4.3	*p* = 0.147
BMI (kg/m^2^)	21.9 ± 2.9	21.6 ± 2.5	*p* = 0.609
Volume of right ovary (mm^3^)	14.5 ± 4.8	12.7 ± 3.5	*p* = 0.096
Volume of left ovary (mm^3^)	13.4 ± 3.7	12.0 ± 4.0	*p* = 0.166
AMH (ng/mL)	9.1 ± 4.0	9.2 ± 3.1	*p* = 0.945
FSH (IU/L)	4.8 ± 1.7	5.5 ± 1.9	*p* = 0.072
LH (IU/L)	7.4 ± 4.5	9.0 ± 4.5	*p* = 0.356
LH/FSH ratio	1.7 ± 1.2	1.8 ± 1.4	*p* = 0.915
Estradiol (pmol/L)	151.6 ± 81.2	138.6 ± 60.2	*p* = 0.709
Progesteron (nmol/L)	4.6 ± 2.2	3.8 ± 2.5	*p* = 0.615
Prolaktin (mIU/L)	289.0 ± 161.3	306.9 ± 181.3	*p* = 0.711
TSH (mIU/L)	2.2 ± 0.8	2.1 ± 0.7	*p* = 0.960
Testosteron (nmol/L)	3.1 ± 1.1	2.9 ± 1.0	*p* = 0.678
Androstenedion (ng/mL)	3.0 ± 1.3	2.9 ± 1.0	*p* = 0.643
DHEAs (μmol/L)	9.3 ± 4.4	9.7 ± 4.9	*p* = 0.114
FAI	8.0 ± 5.1	6.9 ± 4.0	*p* = 0.673
SHBG (nmol/L)	52.9 ± 32.8	52.1 ± 30.8	*p* = 0.742
Fasting insulin (mIU/L)	19.1 ± 8.3	17.5 ± 8.9	*p* = 0.717

**Table 2:** Clinical parameters of the patients after therapy in both groups.

	Gluformin			Inofolic		
Varible	Before Therapy	After Therapy	Statistical Significance	Before Therapy	After Therapy	Statistical Significance
FSH (IU/L)	4.8 ± 1.7	5.5 ± 1.7	*p* = 0.018	5.5 ± 1.9	6.2 ± 1.9	*p* = 0.018
LH/FSH ratio	1.7 ± 1.2	1.2 ± 0.8	*p* = 0.058	1.8 ± 1.4	1.3 ± 1.1	*p* = 0.058
Testosteron (nmol/L)	3.1 ± 1.1	2.0 ± 0.9	*p* < 0.001	2.9 ± 1.0	2.0 ± 1.0	*p* < 0.001
Androstenedion, (ng/mL)	3.0 ± 1.3	2.1 ± 0.6	*p* < 0.001	2.9 ± 1.0	2.3 ± 0.7	*p* < 0.001
DHEAs (μmol/L)	9.3 ± 4.4	7.1 ± 4.2	*p* = 0.001	9.7 ± 4.9	8.6 ± 3.3	*p* = 0.001
FAI	8.0 ± 5.1	3.6 ± 2.1	*p* < 0.001	6.9 ± 4.0	3.6 ± 2.4	*p* < 0.001
SHBG (nmol/L)	52.9 ± 32.8	69.3 ± 40.5	*p* = 0.001	52.1 ± 30.8	73.6 ± 56.4	*p* = 0.001
Fasting insulin, (mIU/L)	19.1 ± 8.3	12.9 ± 4.9	*p* < 0.001	17.5 ± 8.9	13.2 ± 4.0	*p* < 0.001

## Data Availability

Data are contained within the aricle.
